# STIM1 deficiency is linked to Alzheimer’s disease and triggers cell death in SH-SY5Y cells by upregulation of L-type voltage-operated Ca^2+^ entry

**DOI:** 10.1007/s00109-018-1677-y

**Published:** 2018-08-07

**Authors:** Carlos Pascual-Caro, Maria Berrocal, Aida M. Lopez-Guerrero, Alberto Alvarez-Barrientos, Eulalia Pozo-Guisado, Carlos Gutierrez-Merino, Ana M. Mata, Francisco Javier Martin-Romero

**Affiliations:** 1 Department of Biochemistry and Molecular Biology, School of Life Sciences and Institute of Molecular Pathology Biomarkers, University of Extremadura, Avenida de Elvas s/n, 06006 Badajoz, Spain; 2 Applied Bioscience Facility, University of Extremadura, Avenida de Elvas s/n, 06006 Badajoz, Spain; 3 Department of Cell Biology, School of Medicine and Institute of Molecular Pathology Biomarkers, University of Extremadura, Avenida de Elvas s/n, 06006 Badajoz, Spain

**Keywords:** Alzheimer’s disease, Calcium, CRISPR, STIM1, SH-SY5Y, VOCC

## Abstract

**Abstract:**

STIM1 is an endoplasmic reticulum protein with a role in Ca^2+^ mobilization and signaling. As a sensor of intraluminal Ca^2+^ levels, STIM1 modulates plasma membrane Ca^2+^ channels to regulate Ca^2+^ entry. In neuroblastoma SH-SY5Y cells and in familial Alzheimer’s disease patient skin fibroblasts, STIM1 is cleaved at the transmembrane domain by the presenilin-1-associated γ-secretase, leading to dysregulation of Ca^2+^ homeostasis. In this report, we investigated expression levels of STIM1 in brain tissues (medium frontal gyrus) of pathologically confirmed Alzheimer’s disease patients, and observed that STIM1 protein expression level decreased with the progression of neurodegeneration. To study the role of STIM1 in neurodegeneration, a strategy was designed to knock-out the expression of *STIM1* gene in the SH-SY5Y neuroblastoma cell line by CRISPR/Cas9-mediated genome editing, as an in vitro model to examine the phenotype of STIM1-deficient neuronal cells. It was proved that, while STIM1 is not required for the differentiation of SH-SY5Y cells, it is absolutely essential for cell survival in differentiating cells. Differentiated STIM1-KO cells showed a significant decrease of mitochondrial respiratory chain complex I activity, mitochondrial inner membrane depolarization, reduced mitochondrial free Ca^2+^ concentration, and higher levels of senescence as compared with wild-type cells. In parallel, STIM1-KO cells showed a potentiated Ca^2+^ entry in response to depolarization, which was sensitive to nifedipine, pointing to L-type voltage-operated Ca^2+^ channels as mediators of the upregulated Ca^2+^ entry. The stable knocking-down of *CACNA1C* transcripts restored mitochondrial function, increased mitochondrial Ca^2+^ levels, and dropped senescence to basal levels, demonstrating the essential role of the upregulation of voltage-operated Ca^2+^ entry through Ca_v_1.2 channels in STIM1-deficient SH-SY5Y cell death.

**Key messages:**

- STIM1 protein expression decreases with the progression of neurodegeneration in Alzheimer’s disease.
- STIM1 is essential for cell viability in differentiated SH-SY5Y cells.
- STIM1 deficiency triggers voltage-regulated Ca^2+^ entry-dependent cell death.
- Mitochondrial dysfunction and senescence are features of STIM1-deficient differentiated cells.

**Electronic supplementary material:**

The online version of this article (10.1007/s00109-018-1677-y) contains supplementary material, which is available to authorized users.

## Introduction

STIM1 is a 685-aminoacid type I transmembrane protein located in the endoplasmic reticulum (ER), with an essential role in Ca^2+^ mobilization and signaling. The Ca^2+^-sensitive EF-hand domain at the N-terminus rules the conformational change in STIM1 when the intraluminal Ca^2+^ concentration drops below a threshold, and this change triggers the clustering of STIM1 in plasma membrane-ER juxtapositions where it binds store-operated Ca^2+^ channels in order to activate the Ca^2+^ influx pathway termed store-operated Ca^2+^ entry (SOCE) (reviewed in [1]). Therefore, STIM1 is critical as a regulator of Ca^2+^ mobilization, and its role has been studied in a number of cellular events, including gene expression through the calcineurin/NFAT or the calmodulin/CaMKII pathways (reviewed in [2]), and cell proliferation [3], migration, and metastasis [4–8].

Although the role of STIM1 in neuronal function is still poorly understood, there is growing evidence for a role of STIM1 in brain development and function. Early reports described the pattern of STIM1 expression in brain and its activation upon depletion of intracellular Ca^2+^ stores [9, 10]. It was later demonstrated that STIM1, together with dOrai1 (a store-operated Ca^2+^ channel activated by STIM1) were required for the normal activation of motoneurons in *Drosophila melanogaster*, suggesting that replenishment of intracellular Ca^2+^ stores is essential for neuronal function [11]. A significant contribution was the finding that STIM1 inhibits voltage-operated calcium channels (VOCC), such as Ca_v_1.2 [12, 13]. This inhibition is mediated by the same STIM1 domain that activates SOCs, the STIM1-ORAI1 activating region (SOAR), leading to the conclusion that STIM1 reciprocally activates SOCs and inhibits VOCCs. This inhibition is due not only to direct binding of STIM1 to VOCCs but also to enhancing internalization of the channel from the plasma membrane [12].

In addition to Ca_v_1.2, Ca_v_3.1 has been shown to be inhibited by STIM1. In cardiomyocytes, the knocking-down of STIM1 expression increased the current density of T-type voltage-operated Ca^2+^ channels, a result that was explained by the fact that STIM1 also inhibits Ca_v_3.1 by direct interaction and by reducing the surface expression of T-type VOCCs in HL-1 cells [14].

Furthermore, STIM1 has been studied as a possible regulator of differentiation and axonal guidance. For instance, STIM1 is actively recruited to the turning side of the growth cone in response to brain-derived neurotrophic factor (BDNF) [15], and SOCE regulates Ca^2+^ signaling and guidance responses in *Xenopus* growth cones [16]. More recently, it was reported that mGluR1-dependent synaptic potentials are strongly attenuated in the absence of STIM1, and that STIM1 depletion in Purkinje cells impairs cerebellar motor coordination [17]. On the contrary, transgenic mice overexpressing STIM1 exhibited an improvement in contextual learning, with a significant alteration of metabotropic glutamate receptor signaling [18].

Given this collection of evidence, it would not be surprising if STIM1 deficiency were associated with a number of pathologies. In this regard, the presenilin-1 (PSEN1)-associated γ-secretase interacts with STIM1 in human neuroblastoma SH-SY5Y cells, familial Alzheimer’s disease (FAD) patient skin fibroblasts, and mouse primary cortical neurons [19]. Even more interestingly, STIM1 is cleaved at the transmembrane domain, where STIM1 shows a target sequence for γ-secretase, which is shared by the amyloid precursor protein (APP). Indeed, neurons expressing mutant PSEN1 show reduced SOCE and deterioration of dendritic spines [19]. Most AD cases, however, are sporadic or late-onset. There is consensus that apolipoprotein E, epsilon 4 allele (APOE4) is the major risk factor for sporadic early and late-onset forms of AD (reviewed elsewhere [20]). Nevertheless, increasing evidence supports a central role of Ca^2+^ in neurodegenerative processes including AD [21–23], and a review of the “Calcium Hypothesis of Alzheimer’s disease and brain aging” has recently been updated [24] due to the growing evidence linking intracellular Ca^2+^ perturbation with neurodegeneration. Besides, there has been shown to be a Ca^2+^-dependent dysregulation of the high affinity Ca^2+^ transporter plasma membrane Ca^2+^-ATPase in AD brains and its inhibition by the amyloid-β peptide (generated by aberrant cleavage of APP) and tau, the main components of the two major pathological hallmarks of AD [25–27]. Also, a role has been reported for PSENs in Ca^2+^ signaling via modulation of the sarco(endo)plasmic reticulum Ca^2+^-ATPase [28]. The molecular mechanism that involves alteration of Ca^2+^ homeostasis with AD is still far from clear, however, mainly due to the lack of a model system that recapitulates Ca^2+^ dysregulation in neurodegeneration in the absence of mutations in PSEN1, PSEN2, and APP, as occurs in late-onset AD. It is known though that SOCE is decreased and STIM1 and ORAI1 expression are downregulated in rat hippocampal neurons after long-term culturing, an effect that ends up with excessive Ca^2+^ overloading in the ER and increased Ca^2+^ uptake by mitochondria, results that might mimic in vivo neuronal aging [29]. In addition, it has been shown that APP-deficient cells exhibit elevated resting Ca^2+^ concentration within the ER and delayed translocation of STIM1 to ORAI1 upon ER Ca^2+^ store depletion [30].

Human neuroblastoma SH-SY5Y cells have been used for many of the reports referred to above as they provide a model for studying nerve cells, especially when neuritogenesis is stimulated by widely used methods based on different neurotrophic factors, such as BDNF or growth differentiation factor (GDNF). In addition, SH-SY5Y cells express multiple Ca_v_ channels and auxiliary subunits [31], making this cell line a suitable model for the study of the impact of STIM1 on neuronal Ca^2+^ signaling.

In this report, we analyze STIM1 protein expression levels in human brain tissues affected by increasing neurodegeneration associated with sporadic AD, as well as in unaffected age-matched controls. The significant decline in STIM1 expression observed in higher Braak stages led us to study the role of STIM1 in SH-SY5Y cell differentiation triggered by retinoic acid (RA) and BDNF, as well as Ca^2+^ mobilization and mitochondrial function. We generated STIM1-deficient SH-SY5Y cells using a CRISPR-based method to edit the *STIM1* gene locus. Using these STIM1-KO cells, we found that STIM1 is not required for differentiation but is absolutely essential for cell survival in differentiating cells. In these cells, STIM1 loss triggers mitochondrial depolarization and senescence, two features of cell death not previously reported for STIM1-deficient cells. Finally, our results prove that the upregulation of Ca^2+^ entry through Ca_v_1.2 channels leads to cell death, and that stable knocking-down of *CACNA1C* gene transcripts restores the mitochondrial function and senescence to basal levels.

## Methods

### Chemicals

SH-SY5Y cells were purchased from European Collection of Cell Cultures (ECACC) and distributed by Sigma-Aldrich (St. Louis, MO, USA); Phoenix-AMPHO HEK293 were from American Type Culture Collection (ATCC); *all-trans*-retinoic acid, human brain-derived neurotrophic factor (BDNF), collagen (type I), Dulbecco’s modified Eagle’s medium (DMEM), DMEM:F-12 Ham medium, and nifedipine were purchased from Sigma-Aldrich (St. Louis, MO, USA); fura-2-acetoxymethyl ester (fura-2-AM) and BTP2 were from Merck Millipore (Darmstadt, Germany); thapsigargin (Tg) was from Abcam Biochemicals (Cambridge, UK); ML 218 and ω-conotoxin MVIIC were from Tocris Bioscience (Bristol, UK); rhodamine 123, tetramethylrhodamine methyl ester (TMRM), Hoechst 33258, Hoechst 33342, and 5-dodecanoylaminofluorescein di-β-d-galactopyranoside (C12FDG) were from Thermo Fisher Scientific (Waltham, MA, USA); Clarity Max™ Western ECL substrate was from Bio-Rad (Hercules, CA, USA).

### Human tissue selection

Human tissues (medium frontal gyrus) were supplied by the Netherlands Brain Bank (NBB, Amsterdam, The Netherlands) and selected from six individuals diagnosed as having AD (Braaks stages IV, V and VI, ages 84 ± 7) and from six age-matched patients with clinically-diagnosed non-AD degenerative conditions (Braak stage I). Procedures, information – and consent forms of the NBB have been approved by the Medical Ethics Committee of the Vrije Universiteit Amsterdam Medical Centre.

### Antibodies

The rabbit polyclonal anti-STIM1 antibody (raised against the C-terminus) was from ProSci Inc. (Poway, CA, USA), and the mouse monoclonal anti-STIM1 antibody (raised against the N-terminus) was from BD Biosciences (Franklin Lakes, NJ, USA); the mouse monoclonal anti-tubulin beta 3, class III (TUBB3), and the mouse anti-beta tubulin (clone TUB 2.1) were from Sigma-Aldrich (St. Louis, MO, USA); the mouse monoclonal p21 antibody (p21CIP1) and the mouse monoclonal anti-GAPDH antibody were from Santa Cruz Biotechnology (Heidelberg, Germany). Secondary horseradish peroxidase (HRP)-labeled antibodies were from Pierce (Thermo Fisher Scientific, Waltham, MA, USA), or from Santa Cruz Biotechnology (Heidelberg, Germany).

### DNA constructs, transfection, and retroviral infection

DNA constructs for the generation of STIM1-KO (constructs DU52282, DU52301) were described and validated in previous reports by our group [5, 32], and they can be requested on the reagents website https://mrcppureagents.dundee.ac.uk/. Transfection of cells with these DNA constructs was performed with 1–2 μg plasmid DNA per 10-cm dish and polyethylenimine (Polysciences Inc., Eppelheim, Germany) in serum-containing medium.

The construct with the 29mer shRNA cloned into the pRFP-C-RS plasmid to knock-down *CACNA1C* gene expression was purchased from OriGene (#TF314247-A). In this case, retroviral infection and production were performed as described previously [33]. Briefly, Phoenix amphotropic retroviral packaging cells were transfected (10–12 μg per 10-cm dish) with the pRFP-C-RS plasmid. At 24 and 48 h post-transfection, SH-SY5Y cells were incubated with the virus-containing medium with 4 μg/ml polybrene. The culture was extended for an additional of 48 h, and puromycin selection (2 μg/ml) was performed for 5–6 days.

The construct pcDNA-4mtD3cpv to measure mitochondrial-free Ca^2+^ concentration was a gift from Amy Palmer and Roger Tsien (Addgene plasmid # 36324) [34].

### Human brain membranes preparation and immunoblot analysis

Crude human membranes from brain tissues were prepared as described previously [25]. Briefly, tissues were homogenized in 10 mM HEPES/KOH, pH 7.4; 0.32 M sucrose; 0.5 mM MgSO_4_; 0.1 mM phenylmethanesulfonyl fluoride (PMSF); 2 mM 2-mercaptoethanol; and protease inhibitor cocktail solution (Roche Diagnostics, Mannheim, Germany). The homogenates were first centrifuged at 1500 g for 10 min, and the supernatants were further centrifuged at 100,000 g for 45 min. The final pellets were resuspended in 10 mM HEPES/KOH, pH 7.4, 0.32 M sucrose. Protein content was determined using the Coomassie Protein Assay Reagent (Thermo Fisher Scientific).

### Culture and differentiation of SH-SY5Y cells

SH-SY5Y cells were cultured in DMEM with 10% (*v*/*v*) fetal bovine serum (FBS), 2 mM l-glutamine, 100 U/ml penicillin, and 0.1 mg/ml streptomycin in a humidified atmosphere of 95% air/5% CO_2_ at 37 °C. Cell culture dishes and glass coverslips were treated with collagen type I solution (1.5 μg/ml) for a minimum of 30 min at 37 °C prior to cell plating.

For triggering SH-SY5Y differentiation, we followed the protocol described in [35]. Basically, cells were plated at a density of 10^3^–10^4^ cells/cm^2^ onto collagen-coated 24-well plates or 35 mm dishes (Corning Inc., Corning, NY, USA), in DMEM supplemented with 10% FBS. Twenty-four hours after plating, 10 μM *all-trans*-RA was added to the cell cultures, and 2 days later the cells were washed with fresh medium containing 10 μM RA. Six days after the initial plating, the cells were washed and 50 ng/ml BDNF was added to the cell culture which was extended for 2–6 additional days in FBS-free DMEM/F12 medium.

### Generation of genetically modified cells using CRISPR/Cas9 gene editing

CRISPR/Cas9 gene editing was performed as reported previously [5, 32]. Briefly, the guide pair (sense 5′-(G)AGATGACAGACCGGAGTCAT and antisense 5′-(G)AGTCCCTGTCATGGTGGTGT) was identified using the Sanger Institute CRISPR web tool (http://www.sanger.ac.uk/htgt/wge/find_crisprs). This pair targets the exon 5 of the *STIM1* locus (ENSG00000167323), therefore targeting the transcriptional variants NM_001277961.1, NM_001277962.1, and NM_003156.3. The antisense dsDNA guide and the sense guide were cloned into constructs DU52282 and DU52301, as described previously [5, 32]. Transfected cells were selected with puromycin (2 μg/ml) for 48 h, and individual clones were analyzed by immunoblotting and sequencing. Genomic DNA was isolated and the target site was amplified by PCR (primer-fw: 5′-CAAGAGCTAGAAGTGTTCCTGGG; primer-rv: 5′-CTTTGGTTTCCATGGCACAGC). Sequencing of the PCR fragments from the STIM1-KO cells was performed to characterize indels.

### Lysis of cells and immunoblot

Cells were lysed in the following buffer: 50 mM Tris-HCl (pH 7.5), 1 mM EGTA, 1 mM EDTA, 1% (*w*/*v*) Nonidet P40, 1 mM sodium orthovanadate, 50 mM sodium fluoride, 5 mM sodium pyrophosphate, 0.27 M sucrose, 0.1% (*v*/*v*) 2-mercaptoethanol, 1 mM benzamidine, and 0.1 mM phenylmethylsulfonyl fluoride. Clarification of samples was performed after lysis with 0.75–1 ml of ice-cold lysis buffer/dish and centrifugation at 4 °C for 15 min at 20,000 g. Samples were sonicated with five 10-s pulses with a setting of 45% amplitude using a Branson Digital Sonifier. Protein concentration was determined using the Coomassie Protein Assay Reagent.

Lysates (10–40 μg) were subjected to electrophoresis on polyacrylamide gels (4–12% acrylamide) and subsequent electroblotting to nitrocellulose membranes. Membranes were blocked for 1 h at room temperature (RT) in blocking buffer: TBS-T (Tris-buffered saline buffer, pH 7.5, with 0.2% Tween-20) containing 10% (*w*/*v*) non-fat milk. Then the membranes were incubated overnight with the specific antibody diluted in blocking solution at 4 °C, washed, and then incubated with anti-IgG horseradish peroxidase (HRP)-conjugated secondary antibody (typically 1:10,000 dilution) for 1 h at RT. Dilutions of primary antibodies were as follows: anti-STIM1 antibody (1 μg/ml), anti-beta tubulin (clone TUB2.1, 1/3000 dilution), anti-TUBB3 (0.5 μg/ml), anti-p21 (0.8 μg/ml), and anti-GAPDH (0.025 μg/ml). In all cases, luminol substrate was added to the membranes and the signal recorded with the ChemiDoc XRS+ imager (BioRad). The recorded signal was quantified by volumetric integration using ImageJ.

### Assessment of mitochondrial morphology and function

Mitochondrial morphology was evaluated by live imaging of cells stained with rhodamine123 (Rhod123) as described previously [36]. Briefly, cells were incubated with 5 μM Rhod123 for 10 min at 37 °C and then washed in bicarbonate-free Leibovitz’s L-15 medium. Cells were live imaged at 37 °C in an UNO-Okolab stage incubator. Rhod123 was excited with a 465–495 nm excitation filter, and emitted light was detected using a long-pass 515- to 555-nm barrier filter, using an ORCA-EM CCD camera attached to a Nikon Ti-E inverted microscope (Nikon Instruments Europe B.V., The Netherlands).

The mitochondrial inner membrane potential was assessed with TMRM by flow cytometry (FCM) and confocal microscopy. For FCM, detached cells were incubated with 2 nM TMRM in PBS for 30 min at 37 °C. Hoechst 33258 (1 μM) was added to exclude dead cells from analysis. Then, 20,000 cells per sample were acquired using a MACSQuant VYB flow cytometer (Miltenyi Biotech). Kaluza software (Beckman Coulter) was used for data analysis. When confocal microscopy was used, cells were stained with 10 nM TMRM for 30 min at 37 °C. Counterstaining with Hoechst 33342 was performed to facilitate visualization of nuclei. Time-lapse acquisition was performed for 5–10 min, with time intervals of 2 min. In all cases, 10 μM of the mitochondrial uncoupling agent FCCP was used to evaluate the specificity of staining with TMRM. Imaging was done with a FV1000 confocal microscope (Olympus) and fluorescence quantification with FV10 software (Olympus).

The activity of the electron transfer chain complex I was measured at 37 °C as in [37]. The assay buffer was 10 mM Tris–HCl, 50 mM KCl, 1 mM EDTA, and 2 mM KCN (pH 7.4). The quinone CoQ1 (50 μM) was used as electron acceptor. The concentration of cell lysate protein in the assay ranged between 85 and 130 μg/ml. The reaction was initiated by the addition of 75 μM NADH and monitored from the linear decrease of absorbance at 340 nm over 10 min. Then, rotenone (10 μg) was added, and the absorbance at 340 nm was recorded for 10 min. The activity of electron transfer chain complex I was calculated from the difference of the steady-state slope before and after addition of rotenone and expressed in nmol per min per mg of cell lysate protein using an extinction coefficient for NADH of 6.2 mM^−1^ cm^−1^.

### Bright-field microscopy, morphological measurements, and MTT assay

To monitor morphology and measure the length of neurites, cells were fixed with 4% paraformaldehyde in PBS for 15 min at RT and evaluated under bright-field microscopy on a Nikon Ti-E inverted microscope. Measurement of neurites was performed with the NIS-Elements Advanced Research software (Nikon).

Viable cells were estimated by measuring the amount of colored formazan from the reduction of 3-(4,5-dimethylthiazol-2-yl)-2,5-diphenyltetrazolium bromide (MTT) by viable cells as described previously [38, 39].

### Cell cycle distribution and senescence

For cell cycle analysis, detached cells were fixed in cold 70% ethanol for 5 min. After washing in PBS, cells were resuspended in PBS with 0.5% propidium iodide and 1 μg/ml RNAse and incubated in the dark for 2 h with gentle agitation. Cell cycle analysis was performed using a MACSQuant VYB flow cytometer (Miltenyi Biotech), with discrimination of doublets and acquiring > 20,000 cells per sample. To study senescence, detached cells were stained with 1 μM C12FDG in PBS for 30 min at 37 °C in the dark with gentle agitation. After washing in PBS, cells were resuspended in PBS with 1 μM Hoechst 33258 and analyzed by FCM in a MACSQuant VYB flow cytometer (Miltenyi Biotech), acquiring a minimum of 20,000 cells per sample.

### Cytosolic- and mitochondrial-free calcium concentration measurement

Cytosolic-free calcium concentration ([Ca^2+^]_i_) was measured in fura-2-AM-loaded cells as described elsewhere [4, 5, 40]. Excitation fluorescence wavelengths were selected with 340/26 and 387/11 nm filters (Semrock), and emission fluorescence with a 510/10 nm filter. All measurements were performed at 36–37 °C. Excitation/emission conditions were controlled by the NIS-Elements AR software.

Depletion of Ca^2+^ stores was triggered by incubating cells with 1 μM Tg in Ca^2+^-free HBSS with the following composition: 138 mM NaCl; 5.3 mM KCl; 0.34 mM Na_2_HPO_4_; 0.44 mM KH_2_PO_4_; 4.17 mM NaHCO_3_; 4 mM Mg^2+^ (pH = 7.4). SOCE was measured by monitoring the increase of the [Ca^2+^]_i_ after the addition of 2 mM CaCl_2_ to the Tg-containing medium.

[Ca^2+^]_i_ upon depolarizing conditions was measured in Ca^2+^-containing HBSS assay medium (1.26 mM CaCl_2_). To trigger plasma membrane depolarization, HBSS with 90 mM KCl + 5 mM CaCl_2_ was added to cells for 1 min, and then the cells were returned to the initial Ca^2+^-containing HBSS medium with 5.33 mM KCl. When required, Ca^2+^ channel inhibitors were added from a stock solution dissolved in water or DMSO. Calibration of the fura-2 ratio signal was performed by adding 5 μM ionomycin + 5 mM EGTA to cells in Ca^2+^-free HBSS (to assess Rmin), followed by 5 μM ionomycin + 5 mM Ca^2+^ (Rmax). [Ca^2+^]_i_ was calculated as [Ca^2+^]_i_ = (R-Rmin)/Rmax-R) × Kd × (S_f2_/S_b2_), where Sf2 and Sb2 correspond to the emission of fluorescence when the dye is excited at 380 nm under Ca^2+^-free and Ca^2+^-saturating conditions [41]. The fura-2/Ca^2+^ dissociation constant was 224 nM [41], and the ratio S_f2_/S_b2_ was 3.8 in our experimental settings.

Mitochondrial-free calcium concentration ([Ca^2+^]_m_) was measured as described elsewhere [42]. Cells were transiently transfected with the plasmid pcDNA-4mtD3cpv, and 48 h later [Ca^2+^]_m_ was assessed by measuring CFP, YFP, and FRET efficiency between the two channels. Excitation and emission fluorescence wavelengths were selected with the dual CFP/YFP-2 × 2 M-B filter set (Semrock). All measurements were performed at 36–37 °C in Ca^2+^-containing HBSS for 4–5 min. Spectral unmixing (i.e., subtracting the bleedthrough from one channel into another) was performed by determining the bleedthrough coefficients as described in [42]. The background-corrected ratio, i.e., ratio = (FRET_ROI_ − FRET_background_) / (CFP_ROI_ − CFP_bakground_) was converted to [Ca^2+^]_m_ as described in [42], using a dissociation constant for Ca^2+^ = 0.76 mM. The FRET/CFP ratio (*R*) was evaluated after calibrating the signal with the subsequent addition of 5 μM ionomycin + 5 mM EGTA (Rmin), followed by the addition of 5 μM ionomycin + 10 mM Ca^2+^ (Rmax).

### Statistical analysis of data

Statistical analyses between pairs of data groups were done using the Mann-Whitney test of data (non-parametric unpaired *t* test). Analyses were performed with the GraphPad software. Differences between groups of data were taken statistically significant for *p* < 0.05. The *p* values are represented as follows: (*) *p* < 0.05, (**) *p* < 0.01, and (***) *p* < 0.001.

## Results

### STIM1 expression in AD-affected and control human brain tissues

**Fig. 1:**
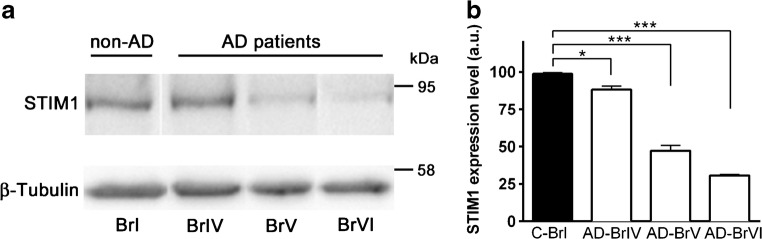
Expression levels of STIM1 in human tissue samples. **a** Samples of medium frontal gyrus membranes (12 μg) from human tissues characterized for diagnosis as non-AD control Braak stage I (first lane) and AD-Braak stages IV, V, and VI, were electrophoresed in a 7.5% SDS-PAGE gel, electrotransferred to nitrocellulose and immunostained with anti-STIM1 antibody. A representative immunoblot from four assays is shown. **b** Quantification of STIM1 protein level relative to β-tubulin is shown as mean ± SE values (a.u., arbitrary units)

As stated above, a higher rate of cleaved STIM1 was found in fibroblasts from patients with PSEN1-mutants associated with FAD [19]. Because no information is available regarding the most frequent form of the disease—the sporadic AD—we monitored total STIM1 levels in human brain tissues (medium frontal gyrus) from patients diagnosed with AD at high Braak stages (IV–VI). Six AD brain samples and six age-matched controls were analyzed. Quantification of STIM1 protein levels, normalized with β-tubulin, showed a significant decrease in total STIM1 for all stages (Fig. 1), and a negative correlation between increasing Braak stage and the STIM1 level, with the later dropping to ~30% at Braak stage VI. This is a novel observation in sporadic AD that fits well with the proposal that STIM1 might be directly involved in the pathogenesis of neurodegenerative diseases. Additional model systems are required, however, to study this possibility in depth.

### STIM1 expression in differentiating SH-SY5Y cells

**Fig. 2:**
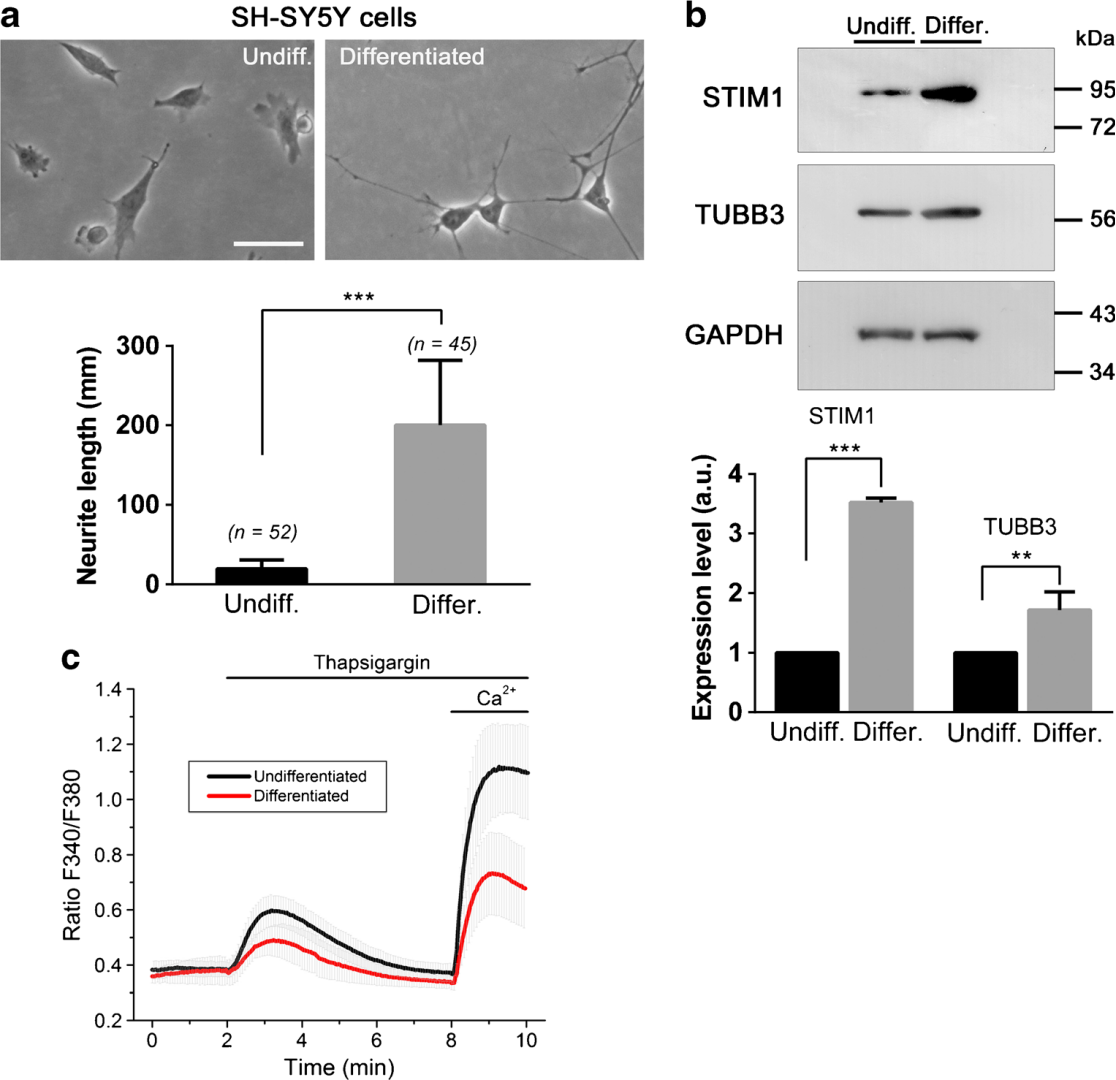
STIM1 expression during differentiation of SH-SY5Y cells. **a** Top: SH-SY5Y cells were differentiated with RA + BDNF, and bright-field microscopy images of cells were recorded (left panel, non-differentiated; right panel, differentiated after 9 DIV). Bottom: Neurite length was measured in undifferentiated cells (*n* = 52), and differentiated cells (*n* = 45), from two independent cultures. Scale bar = 100 μm. **b** Top: Expression of STIM1 and TUBB3 was assessed by immunoblot from undifferentiated cells and cells differentiated after 9–10 DIV with RA + BDNF. Level of GAPDH was assessed as a loading control of the immunoblot. Bottom: The expression of STIM1 and TUBB3 was quantified by immunoblotting with lysates from three independent assays. **c** Store-operated Ca^2+^ entry was evaluated in undifferentiated (black line) and differentiated cells (red line). Fura-2-loaded cells were incubated in a Ca^2+^-free HBSS (assay medium), and 1 μM thapsigargin (Tg) was added to the cells for 6 min. Ca^2+^ (2 mM CaCl_2_) was finally added to the cells to evaluate the extension of Ca^2+^-entry. The experiment was performed at controlled temperature (36–37 °C). Data are presented as the mean ± s.d. of three independent experiments (*n* > 60 cells for each condition)

In order to study the role of STIM1 in neurodegeneration, we followed a well-established protocol to trigger differentiation of SH-SY5Y neuroblastoma cells differentiation in vitro. Cultures were differentiated following the classical protocol with 10 μM retinoic acid (RA) and 50 ng/ml BDNF [35]. Differentiation was evaluated by assessing the level of the neuron-specific β-tubulin 3 (TUBB3) as performed elsewhere [43]. In parallel, we evaluated the length of neurites as a measurement of the level of differentiation (Fig. 2a, b). In both cases, we found the expected increase in TUBB3 expression and neurite length, so this protocol was followed for the differentiation in the subsequent experiments. Using the same cultures, we monitored the level of STIM1 expression and confirmed that there is a marked increase of STIM1 expression in differentiated SH-SY5Y cells. However, this greater expression did not correlate with an increase of SOCE in differentiated cells (Fig. 2c). On the contrary, there was a significant decrease of SOCE suggesting that the monitored upregulation of STIM1 might be related to SOCE-independent events during differentiation. Because our work was focused on the role of decreasing levels of STIM1 in neurodegeneration, we designed a strategy to knock-out *STIM1* gene expression, in order to evaluate the real contribution of STIM1 to cell viability, using SH-SY5Y cells as an in vitro model system.

### Generation of CRISPR/Cas9-mediated STIM1 gene knock-out

**Fig. 3:**
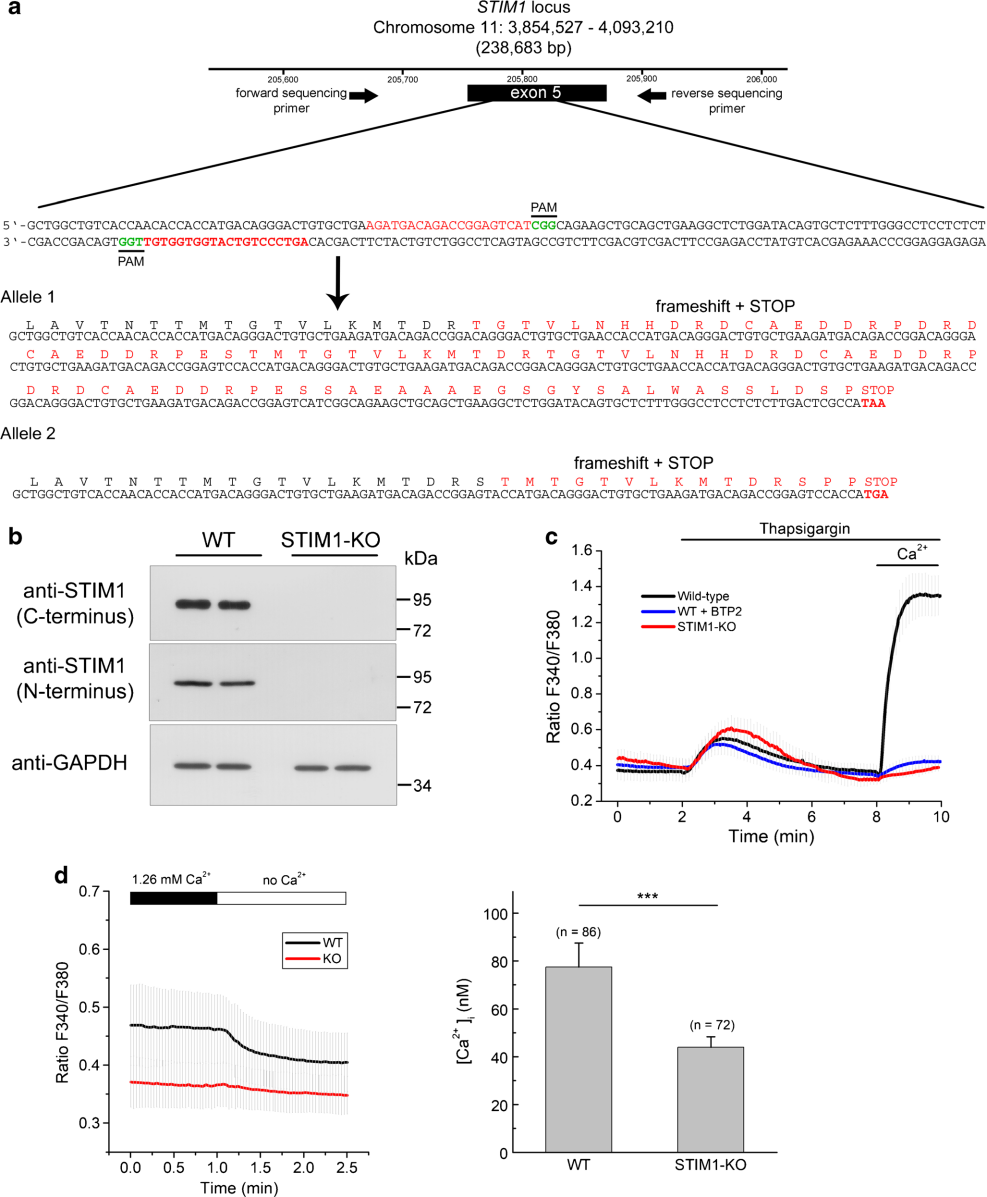
Knockout of STIM1 expression by CRISPR/Cas9 D10A gene editing. **a** Strategy for gene editing using CRISPR/Cas9 D10A in SH-SY5Y cells. A pair of guide RNAs (sense and antisense) was designed to trigger a double nick at exon 5 of the *STIM1* locus. PAM sequences are denoted in green font. Sequencing of a PCR product from the genomic DNA of the selected clone revealed a 211 + 318 base-pair insertion at the target site. The translated protein sequence is denoted in red font, with premature stop codons at the end of the sequences of both alleles. **b** The selected clone of cells was assessed for STIM1 expression by immunoblot, using two different anti-STIM1 antibodies generated against C-terminal and N-terminal epitopes. Anti-GAPDH antibody was used as loading control. **c** Ca^2+^ entry was assessed as in Fig. 2, i.e., triggering the emptying of intracellular stores with 1 μM Tg in Ca^2+^-free HBSS and adding 2 mM Ca^2+^ back to the medium after store emptying. When required, the SOCE inhibitor BTP2 (3 μM) was added together with Tg. Data are presented as the mean ± s.d. of three independent experiments (*n* = 85 cells for KO; *n* = 75 cells for wild-type; *n* = 42 cells for WT + BTP2). **d** Steady-state cytosolic free Ca^2+^ concentration in WT and STIM1-KO cells. Left panel: Fura-2-loaded cells were incubated in Ca^2+^-containing HBSS (1.26 mM), and [Ca^2+^]_i_ was measured as indicated in the Methods section. After recording the F340/F380 ratio signal, the medium was replaced by Ca^2+^-free HBSS to evaluate the contribution of extracellular Ca^2+^ entry to the [Ca^2+^]_i_ in resting conditions. Right panel: After calibration of the fura-2 signal, the [Ca^2+^]_i_ in resting conditions in Ca^2+^-containing HBSS was 77.4 ± 10 nM for wild-type cells, and 44 ± 4.4 nM in STIM1-KO cells. Data are the mean ± s.d. of three independent experiments (*n* = number of cells for each condition)

CRISPR-Cas9 genome editing was used to edit exon 5 in the *STIM1* locus (ENSG00000167323). As our group has reported recently [5, 32], this exon was shared by the known transcriptional variants in *STIM1* gene, and is therefore a preferred target to knock-out the expression of STIM1 proteins. The strategy depicted in Fig. 3a was intended to generate in-frame indels within the target site in order to trigger translational frame shifting. After selecting puromycin-resistant clones, the screening of genomic DNA was performed with flanking primers surrounding the target site. This screening revealed a positive clone with a 211 + 318 bp insertion, therefore leading to premature translational STOP codons in both alleles. Consequently, this clone showed no expression of STIM1 by an immunoblot performed with two different antibodies raised against the N- and the C-termini (Fig. 3b). The lack of STIM1 led to a significant drop in the level of SOCE, which was assessed by the classical Ca^2+^-added back assay after a short incubation with thapsigargin, a sarco(endo)plasmic reticulum Ca^2+^-ATPase inhibitor, in Ca^2+^-free medium. These results confirmed that the selected clone of cells was deficient in this Ca^2+^-influx pathway (Fig. 3c). As a control of this experiment, we measured SOCE in the presence of the SOCE inhibitor BTP2 (also known as YM58483). In parallel, we measured the steady state [Ca^2+^]_i_ in wild-type and STIM1-KO cells in a Ca^2+^-containing assay medium. We found that the absence of STIM1 led to a significant decrease of this value, from 77.4 ± 10 nM for wild-type cells to 44 ± 4.4 nM in STIM1-KO cells (Fig. 3d), suggesting that STIM1 regulates basal [Ca^2+^]_i_.

**Fig. 4:**
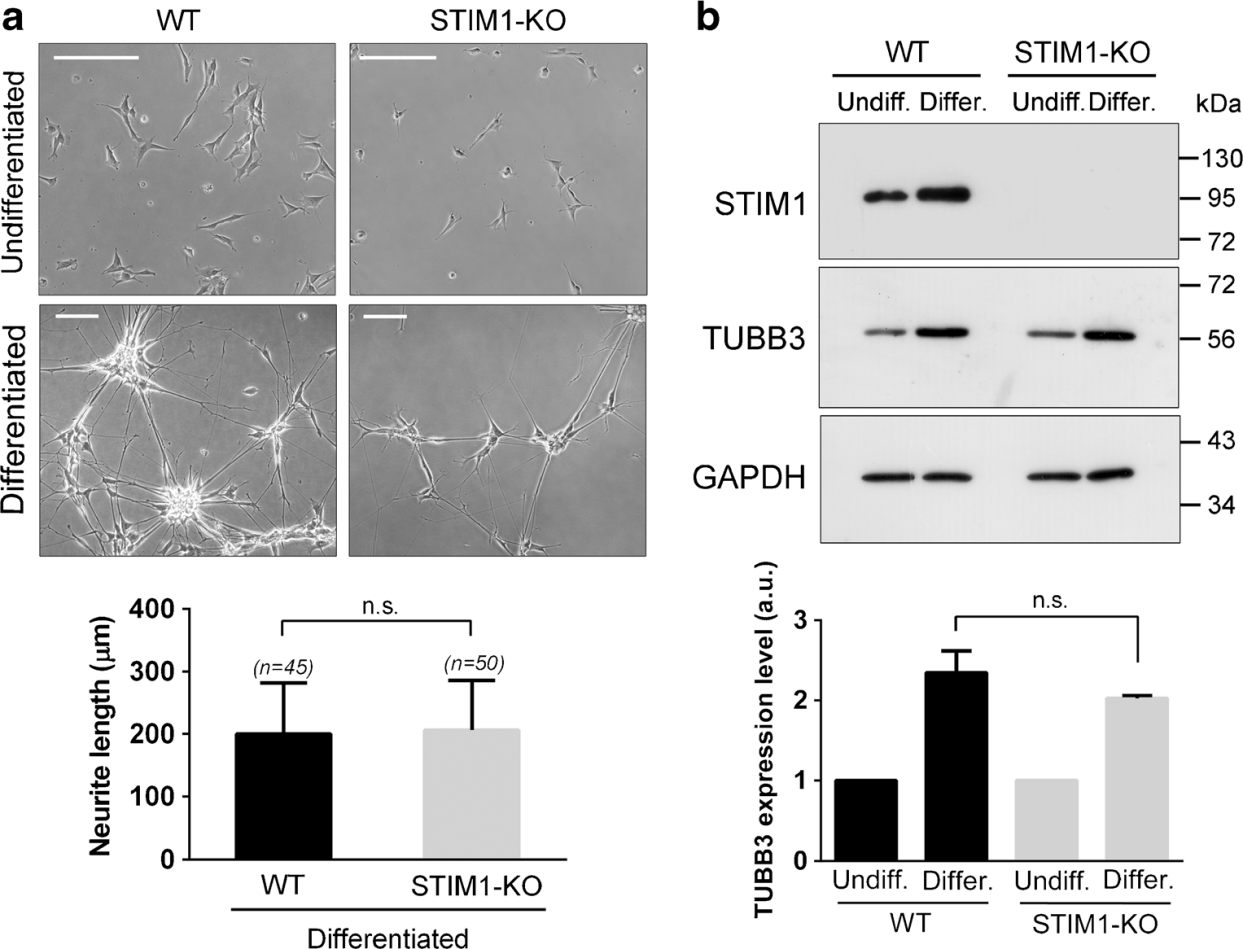
STIM1 deficiency did not modify markers of differentiation. **a** STIM1-KO cells and the parental cell line were differentiated as indicated above, and images of cells in culture were recorded to assess neurite length in undifferentiated cells (top panels), and differentiated after 12 DIV of treatment (bottom panels). Scale bar = 200 μm. Quantification of neurite length revealed no differences between wild-type and STIM1-KO cells. Data are presented as the mean ± s.d. of two independent experiments (*n* = 50 cells for KO; *n* = 45 cells for wt). **b** TUBB3 expression was studied by immunoblot, as in Fig. 2, using GAPDH as a loading control. Differentiation was stopped at 9 DIV and the relative expression of TUBB3 was assessed in three independent experiments (data are the mean ± s.d.)

Then, we proceed to differentiate control SH-SY5Y and STIM1-KO SH-SY5Y cells following the protocol described above. We found that STIM1-KO cells differentiated similarly to the parental cell line. The length of neurites reached similar levels in wild-type and STIM1-KO cells (Fig. 4a). In addition, the assessment of TUBB3 levels by immunoblot showed no statistically significant differences between the two cell lines (Fig. 4b), supporting the conclusion that STIM1 is not required for the full differentiation of SH-SY5Y cells in response to RA + BDNF.

### Loss of STIM1 severely affects cell viability in differentiated cells

**Fig. 5:**
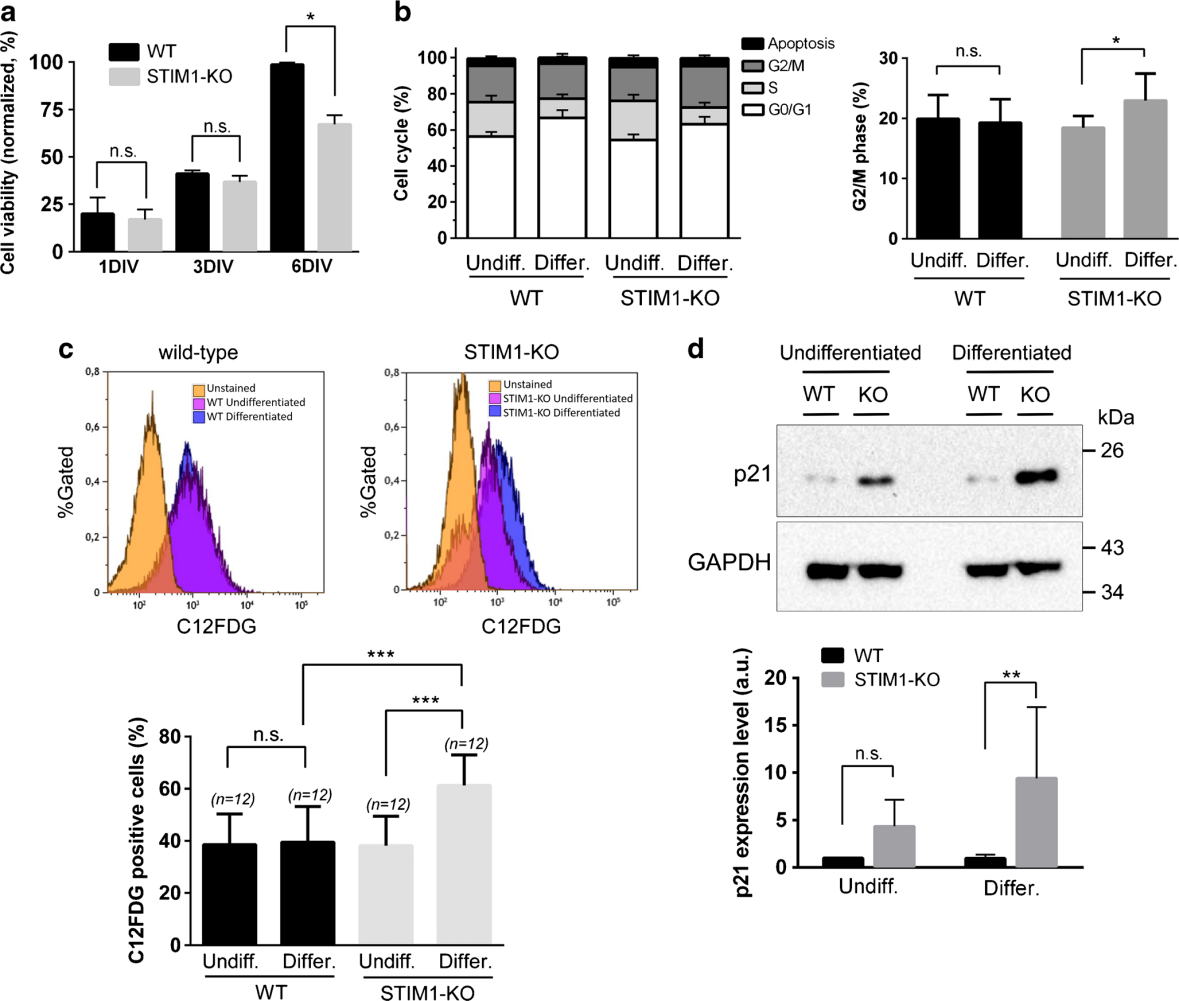
Loss of cell viability in differentiating STIM1-KO cells. **a** Cell viability of wild-type (black bars) and STIM1-KO cells (gray bars) was evaluated with an MTT assay at different stages of differentiation: undifferentiated cells, 24 h in growing medium (1 DIV); 24 h in growing medium + 2 days in differentiating medium (3 DIV); 24 h in growing medium + 5 days in differentiating medium (6 DIV). Data are presented as mean ± s.d. of three independent experiments, and results are normalized to the values obtained from wild-type cells at 6 DIV. **b** The analysis of the cell cycle was performed in undifferentiated (1 DIV) and differentiated cells (6 days of differentiation), and the assay was performed by staining fixed cells with propidium iodide and analyzing cells by flow cytometry. The percentage of cells at G2/M phase is plotted in the right panel to show the statistically significant increase of this phase in STIM1-KO differentiated cells. In both panels, data are the mean ± s.d. of three independent experiments. **c** Cells were cultured as indicated above, and stained with C12FDG to evaluate senescence by flow cytometry. The top panels show representative data histograms, with data from unstained cells as negative control (in orange), undifferentiated cells (pink), and differentiated cells after 6 DIV (blue). The *y*-axis is the normalized cell number, and the *x*-axis is the fluorescence intensity from C12FDG. Data of four independent experiments are shown in the bottom panel as mean ± s.d. **d** Lysates from cells in the experimental conditions described for panels (**b**, **c**) were assessed for p21 expression by immunoblot. The top panel shows a representative blot, with GAPDH as a loading control. The bottom panel shows data of four independent experiments as mean ± s.d.

Although differentiation was unaffected by the loss of STIM1, a drop in cell viability was observed in STIM1-KO cells, a drop which was statistically significant for differentiated cells (Fig. 5a). To study this cell death in depth, we examined whether an alteration of cell cycle underlay the effect on cell viability. The results indicated that there were no significant differences in the cell cycle distribution between undifferentiated wild-type and STIM1-KO cells (Fig. 5b). On the other hand, during differentiation, an accumulation of cells at G0/G1 was observed, a common feature after treatment with RA for other cell types [44, 45]. Although the extent of the increase at G0/G1 was the same in both cell lines, the reduction in the number of cells at S phase was significantly greater in differentiated STIM1-KO cells than in wild-type cells. This latter result also reflects the differential arrest observed at G2/M which was significantly greater for differentiated STIM1-KO cells than for wild-type cells (Fig. 5b), a result that suggests a slowing-down of the cell cycle at this transition in the absence of STIM1. We also measured the level of haplodiploidy as an index of apoptosis, finding no significant differences between wild-type and STIM1-KO cells (data not shown), leading to the conclusion that this pathway has a minimal contribution to the drop in cell viability observed during differentiation.

Because of the increase in the level of cells at G2/M-phase, we tested the hypothesis that the cell death in the STIM1-KO case might be a consequence of an increase in senescence. We therefore measured senescence by incubating cells with 5-dodecanoylaminofluorescein di-beta-d-galactopyranoside (C12FDG), a fluorogenic substrate for the senescence-associated beta-galactosidase activity [46], and measuring the product by flow cytometry. The results indicated a significant increase of cellular senescence in differentiated STIM-KO cells (Fig. 5c), which explains the drop in cell viability shown in Fig. 5a (MTT assay). Although senescence can be triggered by a wide range of stress signals, it is widely accepted that the cyclin-dependent kinase inhibitor 1 (the product of the gene CDKN1A, also known as p21CIP1 or p21) is an essential mediator, and therefore senescent cells show upregulated expression of p21 [47]. This was the case for STIM1-KO cells (Fig. 5d) which showed upregulation of p21 that was statistically significant for differentiated cells.

### Mitochondrial dysfunction in STIM1-KO cells

**Fig. 6:**
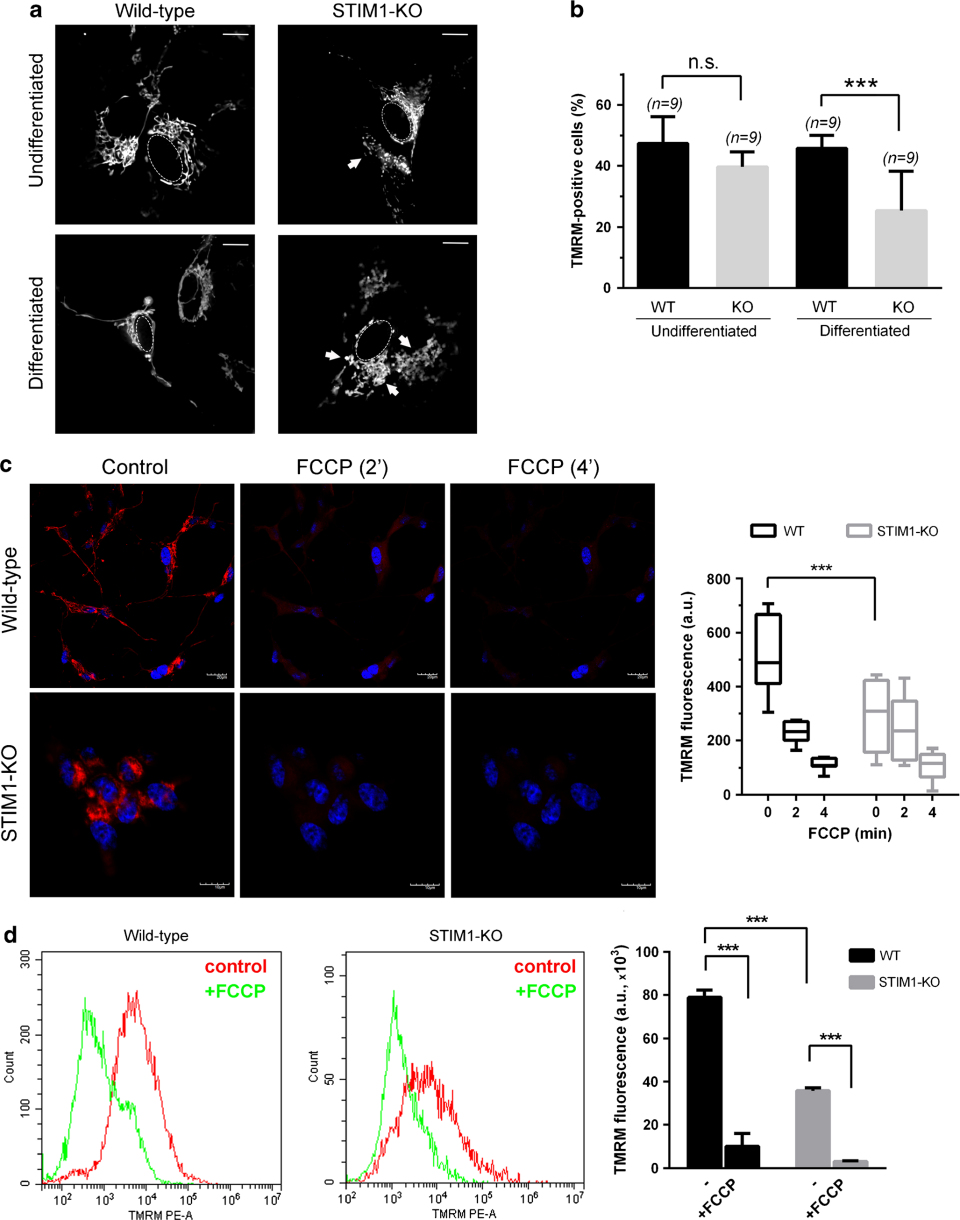
Loss of mitochondrial function in STIM1-KO differentiated cells. **a** Cells, differentiated as indicated in Fig. 2, were stained with rhodamine 123 in phenol red and serum-free medium, and observed under epifluorescence. Samples, in bicarbonate-free Leibovitz’s L-15 medium, were live-cell imaged at 37 °C in an UNO-Okolab stage incubator and analyzed with a Plan Apochromat × 100 (NA 1.45) oil immersion objective. Z-sections (0.2-μm steps) were recorded, and after deconvolution, the total projection was analyzed to observe mitochondria morphology. Representative images from two independent experiments are shown (> 20 cells per condition). Dashed line depicts nuclear envelope. Scale bar = 10 μm. **b** Cells, under the experimental conditions described for panel (**a**), were stained with TMRM, and the fraction of positively TMRM-stained cells was analyzed by flow cytometry. Data are presented as mean ± s.d. of three independent experiments. **c** Cells were cultured on glass coverslips and stained with TMRM. Cells were visualized under confocal microscopy to evaluate the intensity and morphology of staining in resting conditions. FCCP (10 μM) was added to assess non-specific staining and loss of signal due to mitochondrial inner membrane depolarization after 2 and 4 min. Hoechst 33342 was used to stain cell nuclei (blue). Scale bar = 10 or 20 μm, as indicated. TMRM fluorescence intensity from different ROIs was quantified, and the data (mean ± s.d. of two independent experiments) are shown as a box plot (right panel). **d** TMRM-stained cells were analyzed by flow cytometry. Histograms (left panel) depict the number of events (*y*-axis) and fluorescence intensity (*x*-axis) for wild-type and STIM1-KO cells before and 10-min after addition of 10 μM FCCP. Total fluorescence data of two independent experiments are shown in the right panel bar chart as mean ± s.d.

The initial viability test used in this study (MTT assay in Fig. 5a) is based on the activity of dehydrogenases, mostly from mitochondria, so we next tested the hypothesis of a mitochondrial dysfunction triggered by the differentiation in the absence of STIM1. The staining of mitochondria with rhodamine 123 in order to monitor mitochondrial morphology indicated the occurrence of extensive mitochondrial fission in differentiated STIM1-KO cells (Fig. 6a), suggesting the possibility of higher levels of mitochondrial depolarization in these cells. The analysis of the mitochondrial inner membrane polarization was performed with tetramethylrhodamine methyl ester (TMRM). A significant drop in the level of TMRM-positive cells, i.e., with evident mitochondrial depolarization, was found in differentiated STIM1-KO cells (Fig. 6b), indicating a significant loss of mitochondrial function. Undifferentiated cells showed similar TMRM fluorescence levels in both the STIM1-KO and the wild-type cell lines, in agreement with the results of cell viability shown in Fig. 5a (data are shown as 1 DIV), and the senescence data in Fig. 5c. This analysis was also performed by confocal microscopy, and the fluorescence levels were quantified from differentiated wild-type and STIM1-KO cells, an experiment that revealed a drop in ~50% in TMRM fluorescence in STIM1-deficient cells (Fig. 6c). The mitochondrial uncoupler FCCP (10 μM) was added to evaluate the background signal in our experimental setting. Similarly, we assessed TMRM fluorescence in wild-type and STIM1-KO cells by flow cytometry (Fig. 6d), in resting conditions and after 10 min of incubation with 10 μM FCCP. As noted above for confocal microscopy, FCCP was used to evaluate the background signal of TMRM. The results of the experiment confirmed the drop in mitochondrial polarization in STIM1-deficient cells to ~45% of that found for wild-type cells.

**Figure 7:**
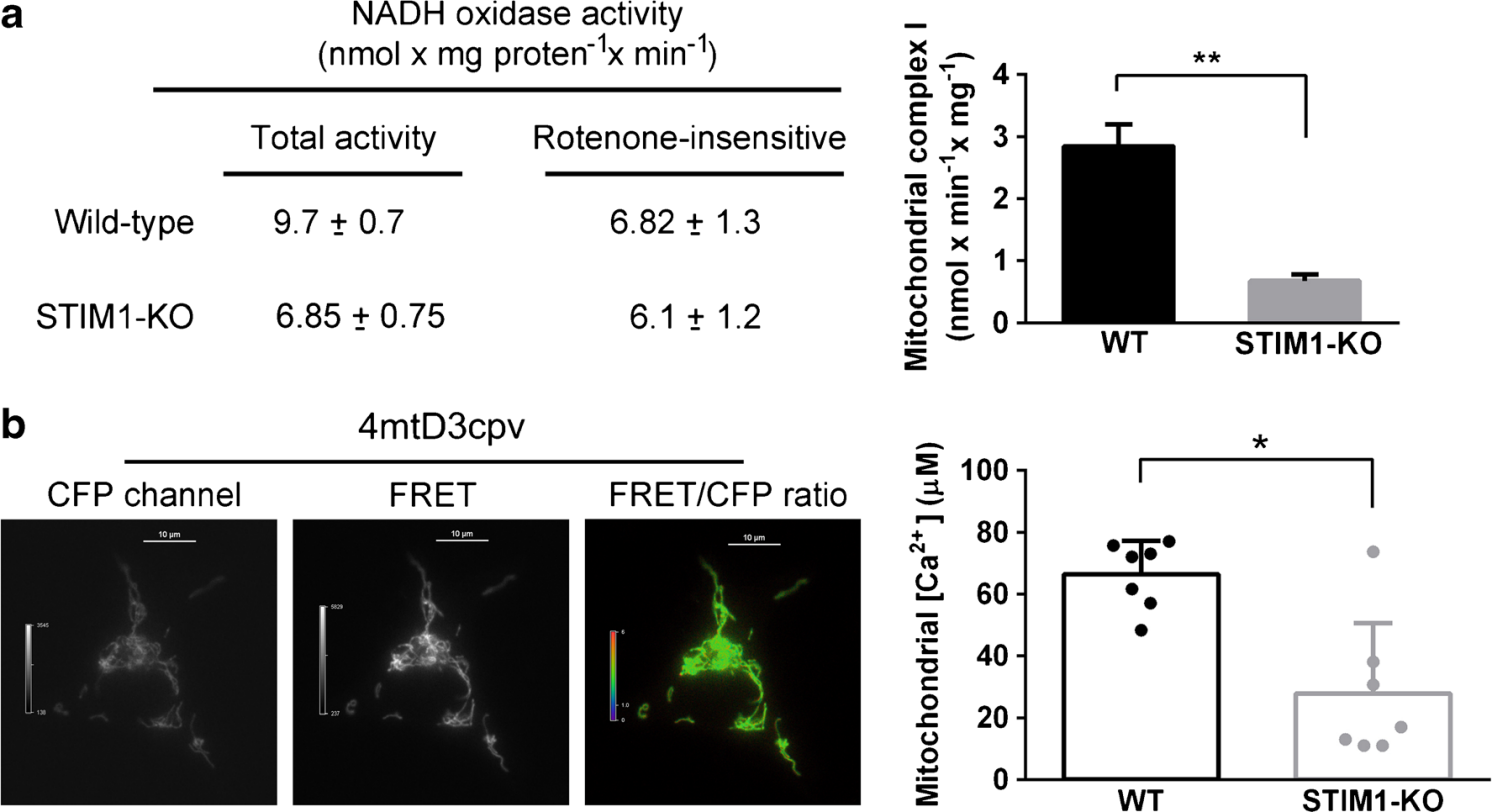
Mitochondrial electron transport complex and mitochondrial Ca^2+^ levels. **a** Total NADH oxidase activity and rotenone-sensitive activity was assessed from differentiated SH-SY5Y cell lysates (WT and STIM1-KO). Data are presented as the mean ± s.d. of two independent experiments. Right panel shows the difference between total activity and the remaining activity after rotenone addition to the assay, i.e., the rotenone-sensitive NADH oxidase. **b** Wild-type and STIM1-KO cells were transiently transfected for the expression of the Ca^2+^ sensor 4mtD3cpv and 48 h later emission of fluorescence was recorded for CFP, FRET (left and middle panels,), and YFP channels to monitor photobleaching. FRET/CFP ratio signal (right panel) was recorded for cells in Ca^2+^-containing HBSS for 4–5 min. Calibration of FRET/CFP ratio to calculate Rmin and Rmax was performed individually for every assay. [Ca^2+^]_m_ data are presented as the mean ± s.d. of seven independent experiments

Because mitochondrial inner membrane polarization relies on efficient electron transport through respiratory complexes, we measured the activity of complex I (NADH-coenzyme Q oxidoreductase) (Fig. 7a), the most important NADH oxidase activity in mitochondria, and found more than 50% decrease of the activity of this complex in STIM1-KO cells. This result demonstrated that deficiency of STIM1 was accompanied by a major impairment of mitochondrial function. The negative charge of the membrane potential established by the respiratory chain drives other important events, such as Ca^2+^ uptake by the mitochondrial Ca^2+^ uniporter (reviewed in [48]). Therefore, we measured the steady-state mitochondrial-free Ca^2+^ concentration ([Ca^2+^]_m_) in resting conditions using the genetically encoded Ca^2+^ sensor 4mtD3cpv (Fig. 7b). The transient transfection of wild-type and STIM1-KO cells with this indicator showed that [Ca^2+^]_m_ in STIM1-deficient cells was ~40% of that found in wild-type cells (27.8 ± 22.8 μM for KO vs 66.4 ± 10.9 μM for WT).

### Contribution of L-type voltage-gated Ca^2+^ entry to cell death in STIM1-KO cells

**Figure 8:**
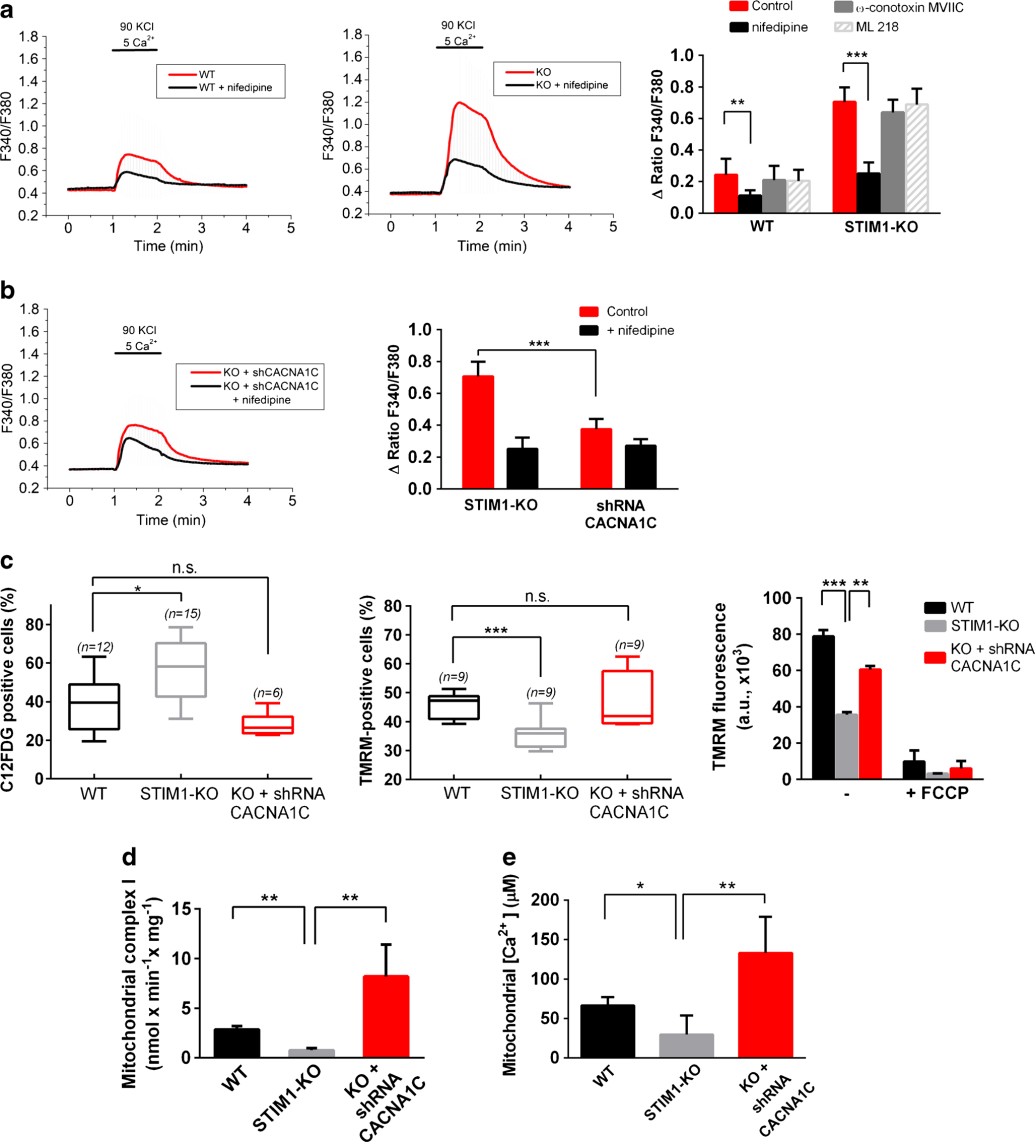
Increased cellular Ca^2+^ influx underlies mitochondrial failure and augmented senescence. **a** Changes in cytosolic-free Ca^2+^ concentration were analyzed in fura-2-loaded cells. Cells in HBSS containing 1.26 mM Ca^2+^ were subjected to 1-min depolarization with 90 mM KCl (red line). CaCl_2_ in the HBSS was increased to 5 mM during depolarization to facilitate the Ca^2+^ influx recording. In parallel experiments, 10 μM nifedipine was added to the assay medium during the recording (black line). Right panel: the increase of the F340/F380 ratio triggered by 90 mM KCl in the presence of VOCCs blockers is shown as mean ± s.d. of 3 experiments (a minimum of 70 cells per experimental condition). Final concentrations: 10 μM nifedipine, 1 μM ω-conotoxin MVIIC, 3 μM ML 218. **b** STIM1-KO cells, or STIM1-KO cells stably expressing a specific shRNA to knock-down *CACNA1C* transcripts, were treated as described in panel (**a**). The left panel shows a representative experiment, and the bar chart of the right panel shows the increase in the F340/F380 ratio evoked by depolarization (mean ± s.d. of two independent experiments; *n* > 60 cells per condition). **c** Senescence (left panel) and mitochondrial polarization (middle and right panels) were assessed from differentiated cells after 6 DIV, staining with C12FDG as described in Fig. 5c and TMRM as in Fig. 6b–d, respectively. Data are mean ± s.d. of three independent experiments (number of replicates is shown for each condition). **d** Rotenone-sensitive NADH oxidase activity was assessed from differentiated SH-SY5Y cell lysates (wild-type, STIM1-KO, and STIM1-KO + shRNA for *CACNA1C*). Data are presented as the mean ± s.d. of two independent experiments. **e** Cell were transiently transfected for the expression of the Ca^2+^ sensor 4mtD3cpv. Mitochondrial [Ca^2+^] was assessed as described in Fig. 7. Data of six independent experiments are shown in the right panel bar chart as mean ± s.d.

In the finding of the molecular mechanism underlying the observed senescence, mitochondrial failure, and cell death triggered by the loss of STIM1, we focused on the possibility of an impairment in Ca^2+^ mobilization. As stated above, STIM1 inhibits Ca_v_1.2 channels, and therefore the dihydropyridine-sensitive voltage-gated Ca^2+^ entry. We therefore hypothesized that STIM1-KO cells might have augmented Ca^2+^ entry through this pathway that would be dysregulating Ca^2+^ homeostasis. To test this hypothesis, we measured Ca^2+^ entry in fura-2-loaded cells in response to a 1-min depolarization with 90 mM KCl. This experiment revealed increased Ca^2+^ entry in response to depolarization with KCl in STIM1-KO cells compared to wild-type cells (Fig. 8a). In order to evaluate the contribution of dihydropyridine-sensitive voltage-gated Ca^2+^ channels (VOCCs) to this increase, we measured cytosolic Ca^2+^ levels in the presence of 10 μM nifedipine, a concentration that blocks Ca^2+^ entry through L-type VOCCs [49, 50], and we found that the Ca^2+^-entry sensitive to nifedipine was ~3-fold greater in STIM1-KO cells than in wild-type cells (Fig. 8a), a result that fits well with the function reported for STIM1 as a negative modulator of VOCCs [12, 13]. We also evaluated the contribution of other VOCCs by studying the effect of well-known inhibitors on the increase of [Ca^2+^]_i_ under depolarizing conditions (90 mM KCl). Neither ω-conotoxin MVIIC, an inhibitor of N-, P-, and Q-type channels, nor ML 218, a highly specific inhibitor of T-type channels, prevented the increase of [Ca^2+^]_i_ in response to 90 mM KCl (Fig. 8a), suggesting that the observed augmented Ca^2+^ influx in STIM1-KO cells was due to L-type Ca^2+^ channels only.

To test whether the upregulation of this Ca^2+^ entry contributed to senescence and loss of mitochondrial inner membrane potential, we knocked-down *CACNA1C* gene expression by stable transfection of a specific short hairpin RNA (shRNA) through retroviral infection of STIM1-KO cells. This long-term silencing of *CACNA1C* gene transcripts reduced Ca^2+^ entry in response to depolarization (Fig. 8b), with the nifedipine-sensitive Ca^2+^ entry being similar to that found in wild-type cells, confirming the functional knock-down of Ca^2+^ influx through L-type VOCCs. As a result, the level of senescence (i.e., C12FDG-positive cells) in differentiated STIM1-KO cells fell back to the background level after silencing Ca_v_1.2 expression (Fig. 8c). Moreover, the mitochondrial inner membrane potential, measured with TMRM, was restored in differentiated STIM1-KO cells when we knocked-down *CACNA1C* gene expression (Fig. 8c). *CACNA1C* silencing also restored mitochondrial complex I activity, which reached values twofold higher than that observed in wild-type cells (Fig. 8d), and [Ca^2+^]_m_ was raised twofold up to 132.9 ± 46 μM when *CACNA1C* gene expression was silenced in STIM1-depleted cells (Fig. 8e). The increase of the [Ca^2+^]_m_ was observed in parallel with a slight raise of the [Ca^2+^]_i_ in CACNA1C-depleted cells (Supp. File 1). However, the [Ca^2+^]_i_ in STIM1-KO cells with silenced CACNA1C (54.3 ± 5.9 nM) was significantly lower than that observed for wild-type cells (80.6 ± 4.9 nM), suggesting that the increase of [Ca^2+^]_m_ was not significantly influenced by the steady-state value of the [Ca^2+^]_i_.

Altogether, these results confirmed that the upregulation of Ca^2+^ transport through L-type VOCCs underlies mitochondrial dysfunction and senescence during differentiation of SH-SY5Y cells in the absence of STIM1.

## Discussion

Early-onset familial Alzheimer’s disease-3 (AD3) is caused by heterozygous mutation in the PSEN1 gene on chromosome 14q24 (OMIM entry #607822, and references therein). PSEN1 and PSEN2 are important determinants of γ-secretase activity, which is responsible for the proteolytic cleavage of the amyloid precursor protein (APP). Moreover, the transmembrane domain of STIM1 is also a target for the PSEN1-associated γ-secretase [19], and it has been reported that fibroblasts from patients with familial AD show decreased levels of total STIM1 [19], and attenuation of SOCE is observed in cells bearing PSEN1 mutations [51]. These observations support a role for Ca^2+^ dysregulation in the pathogenesis of familial AD. However, the more frequent sporadic late-onset AD is genetically complex, and therefore the lack of suitable model animals makes the study of the involvement of Ca^2+^ signaling in this form of the disease more challenging.

In this work, we report for the first time that a decrease of STIM1 content is associated with neurodegeneration in sporadic AD human brain. Thus, this type of study of cell behaviour with decreased STIM1 levels is an experimental approach to understanding the role of Ca^2+^ signaling and Ca^2+^ homeostasis dysregulation in neurological diseases. In this regard, such genome editing tools as CRISPR/Cas9 provide the opportunity to knockout specific genes with a minimal impact on the genome, i.e., without triggering genome instability. To this end, we used a nickase version of the Cas9 nuclease, a strategy that reduces the number of off-targets because it requires two independent guide RNAs to define the target sequence to be edited. This approach, which had been successfully developed in our laboratory for other cell types [5, 32], resulted in the loss of STIM1 protein expression in SH-SY5Y cells by the insertion of in-frame premature STOP codons. With STIM1-deficient cells, we demonstrate here that STIM1 is not directly involved in the differentiation of neuroblastoma SH-SY5Y cells triggered by RA + BDNF, although STIM1 is essential to sustain cell viability in differentiated cells. Differentiated SH-SY5Y cells constitute a model with which to study neuronal function in vitro, and therefore the fact that STIM1 is fully required to sustain the viability of differentiated SH-SY5Y cells raises multiple questions regarding the signaling pathways controlled by STIM1 in neurons.

The results shown in this report point to mitochondria function as a target of Ca^2+^ dysregulation triggered by the absence of STIM1 in SH-SY5Y cells, similarly to what it has been described for fibroblasts from patients with loss-of-function mutations in *ORAI1* or *STIM1* loci [52]. These fibroblasts showed reduced expression of mitochondrial complexes CI and CIV and supercomplex CICIII2, as well as reduced proton pumping rate by the electron transport chain, limiting the mitochondrial membrane potential. Similarly, a significant reduction in membrane potential has been observed in this report for STIM1-KO cells using the potential-sensitive dye TMRM. Ca^2+^ also regulates the activity of three enzymatic reactions of the citric acid cycle: isocitrate dehydrogenase, α-ketoglutarate dehydrogenase, and pyruvate dehydrogenase, which is activated by the Ca^2+^-sensitive pyruvate dehydrogenase phosphatase (reviewed in [53]). Here, we show that mitochondrial-free Ca^2+^ levels are significantly reduced in STIM1-KO cells, in contrast to what it has been reported in STIM1-deficient mouse embryonic fibroblasts using Rhod-2-AM [54]. However, Ca^2+^ measurements obtained with Rhod-2 are not accurate and the results obtained with this dye should be taken with care, because they have a response to dynamic [Ca^2+^] changes qualitatively different to other Ca^2+^ sensors, probably due to the generation of Rhod-2 derivatives during light excitation [55]. Here, we report absolute [Ca^2+^]_m_ levels thanks to the use of the genetically encoded Ca^2+^ sensor 4mtD3cpv, that bypasses the methodological problems observed with Rhod-2. Because Ca^2+^ stimulates the citric acid cycle, the drop in [Ca^2+^]_m_ monitored in STIM1-KO cells would limit the NADH/FADH_2_ production. Together with this limited electron donor supply, the inhibition of the complex I shown in Fig. 7 explains well the mitochondrial inner membrane depolarization in STIM1-KO cells, an event that risks cell viability.

It is also important to consider that the increasing levels of STIM1 observed during differentiation were not accompanied by any increase in SOCE (see Fig. 2b, c). On the contrary, it is remarkable that the increase of STIM1 in differentiated cells is associated with a striking decrease of SOCE, suggesting a non-canonical function of STIM1 in fully differentiated neurons. Additional roles for STIM1 in fully differentiated cells have been proposed, such as the control of neuronal excitability and the intrinsic plasticity of Purkinje neurons [56] in agreement with the results from transgenic mice overexpressing STIM1 which show improved contextual learning [18]. Another role for STIM1 is the regulation of other non-SOC Ca^2+^ channels, such as the voltage-operated Ca^2+^ channels (VOCCs), Ca_v_1.2 or Ca_v_3.1, but STIM1 has also been implicated in the regulation of the glutamate ionotropic receptor AMPA type subunits 1/2 [57], and the glutamate metabotropic receptor 1 (mGluR1) [17]. With regard to this set of channels and receptors, it is known that STIM1 positively modulates glutamate receptors. However, the molecular mechanism for the regulation of mGluR1 and GluA1/2 remains to be elucidated. On the contrary, it has been proposed that STIM1 downregulates voltage-operated Ca^2+^ entry mainly by inhibition of the Ca_v_1.2 channel and triggering its internalization [12, 13]. Our results support the conclusion that STIM1 is a negative regulator of Ca_v_1.2, and predict an augmented Ca^2+^ entry through L-type VOCCs in those (patho-)physiological conditions that develop with decreasing levels of STIM1. This appears to be the case in AD according to recent findings with familial AD patient fibroblasts [19] and the present findings for sporadic AD. In addition, an elevated VOCC activity has been recorded in CA1 neurons from hippocampal slices in aged rats, an alteration that negatively regulates short-term neuronal plasticity [58]. In this regard, compared with wild-type mice, transgenic mice designed to develop accumulation of amyloid β-peptide and selective hyperphosphorylated tau pathology in hippocampal CA1 neurons (3 × TgAD mice) showed age-related elevation of L-type Ca^2+^ channel current density in these specific neurons, suggesting a contribution of VOCCs to the selective vulnerability of CA1 neurons to tau pathology, and a possible role to neuronal degeneration in AD patients [59]. Accordingly, the long-term treatment of patients with blockers of L-type VOCCs reduced the incidence of dementia by 50% [60], further confirming earlier observations that had associated hypertension with an increased risk of both vascular dementia and AD [61]. Similarly, the prolonged treatment with the VOCC blocker nimodipine ameliorated age-related memory impairment in rats [62], and isradipine attenuated β-amyloid oligomer toxicity by reducing Ca_v_1.2 expression in vitro and inhibiting Ca^2+^ influx [63], supporting the feasibility of a therapeutic approach with dihydropyridines in AD patients (reviewed in [64]).

Because a continuing decline of STIM1 levels is observed in aged neurons [29], and also during neurodegeneration in sporadic AD patients as reported here, the loss of STIM1 could be taken into consideration to explain the pathogenesis of neurological diseases. Our results demonstrate that the loss of STIM1 triggers cell death by upregulation of Ca^2+^ entry through VOCCs. The silencing of a specific gene transcript (*CACNA1C* for Ca_v_1.2 channel) prevents the senescence triggered by the absence of STIM1, restores the basal levels of mitochondrial inner membrane potential and the mitochondrial respiratory complex I activity in STIM1-deficient cells, fully supporting a causal link between STIM1 reduction and cell death. Altogether, the results prove that Ca_v_1.2 upregulation is essential to trigger cell death in STIM1-deficient cells, a proposal that fits well with the protective effect of dihydropyridines against neurodegeneration in AD patients.

## Conclusions

Because STIM1 is a key regulator of Ca^2+^ channels, the observation that there is a significant loss of STIM1 in hippocampal human tissue from AD patients supports a role for Ca^2+^ transport and signaling dysregulation in AD. Our results demonstrate that one of the most important downstream targets for the absence of STIM1 in differentiated neuroblastoma cells is the upregulation of Ca^2+^ influx through Ca_v_1.2, which fully explains the Ca^2+^ dysregulation observed in hippocampal neurons from model animals as well as the protective effect of VOCCs inhibition in AD patients. In this regard, STIM1-KO cells constitute an in vitro model with which to study the pathogenesis of the disease, and will be useful to help understand the role of STIM1 in the modulation of additional channels and pathways involved in neurodegeneration, with a possible impact on the search for new therapeutic targets.

## Electronic supplementary material

ESM 1 (PNG 2673 kb)

## Abbreviations

AD: Alzheimer’s disease
BDNF: Brain-derived neurotrophic factor
C12FDG: 5-Dodecanoylaminofluorescein di-β-d-galactopyranoside
ER: Endoplasmic reticulum
FCCP: Carbonyl cyanide 4-(trifluoromethoxy)phenylhydrazone
RA: Retinoic acid
SOC: Store-operated Ca^2+^ channel
SOCE: Store-operated Ca^2+^ entry
TMRM: Tetramethylrhodamine methyl ester
Tg: Thapsigargin
VOCC: Voltage-operated Ca^2+^ channel
