# Supplementary figures and images for: STIM1 deficiency is linked to Alzheimer’s disease and triggers cell death in SH-SY5Y cells by upregulation of L-type voltage-operated Ca2+ entry
Source: J Mol Med (Berl). 2018 Aug 7;96(10):1061–79. doi: 10.1007/s00109-018-1677-y (PMC6133163; doi:10.1007/s00109-018-1677-y)

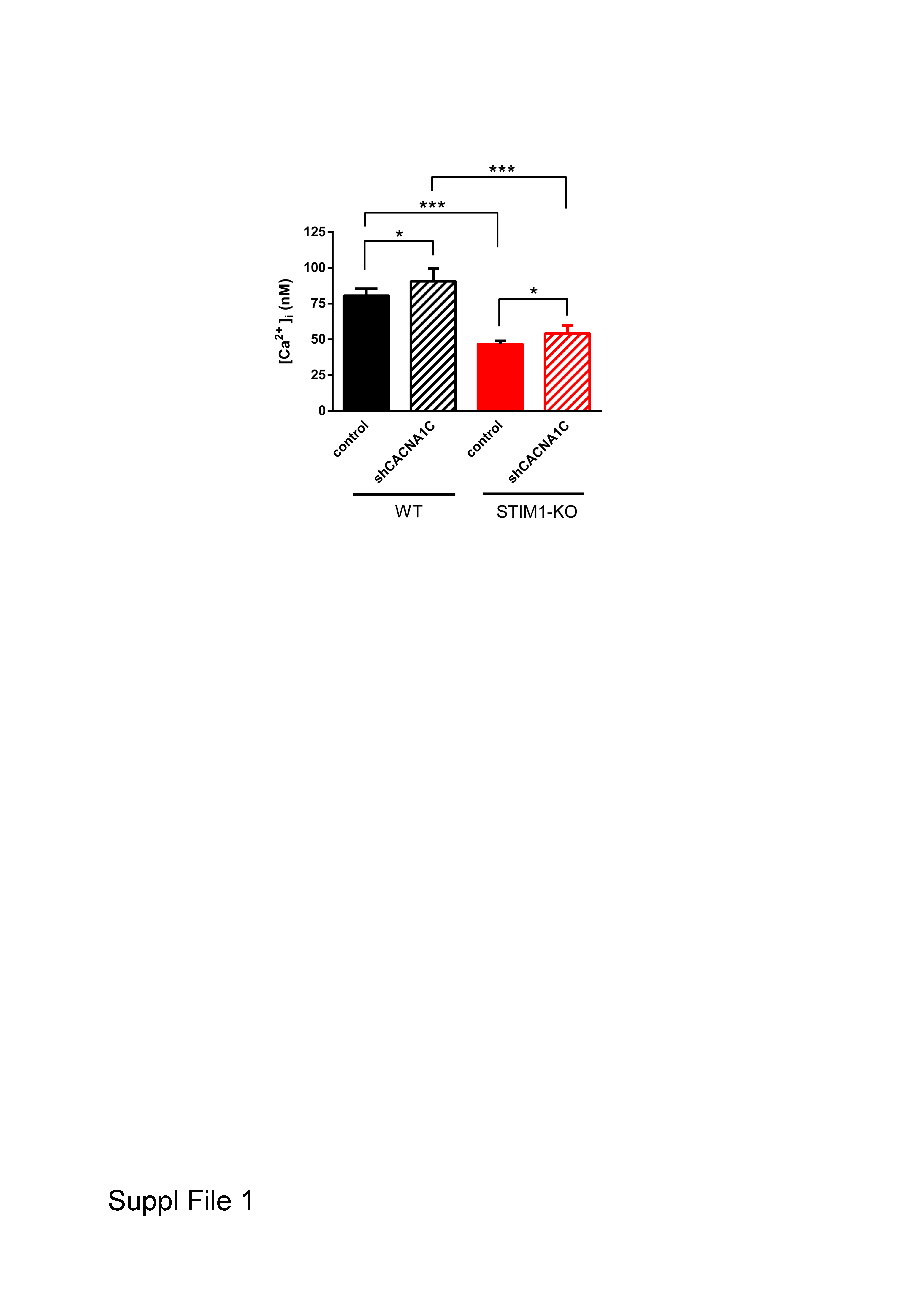

Supplement: Supplementary file 1 — (PNG 2673 kb) [file 109_2018_1677_MOESM1_ESM.png]
